# Supplementary material for: Disentangling the role of Africa in the global spread of H5 highly pathogenic avian influenza
Source: Nat Commun. 2019 Nov 22;10:5310. doi: 10.1038/s41467-019-13287-y (PMC6874648; doi:10.1038/s41467-019-13287-y)
Supplement: Supplementary file 1 — Supplementary Information [file 41467_2019_13287_MOESM1_ESM.pdf]

Disentangling the role of Africa in the global spread of H5 highly pathogenic avian influenza

Fusaro et al.

Supplementary Information

SUPPLEMENTARY METHODS

Datasets design

Global datasets

Data were collected from three HPAI H5 epizootics which occurred in Europe, Asia and Africa between 2005 and 2018 and were caused by clades *2.2* (H5N1), *2.3.2.1c* (H5N1) and *2.3.4.4-B* (H5NX).

All *HA* sequences and relative epidemiological information of the H5N1 and H5NX subtype available in the Global Initiative on Sharing All Influenza Data (GISAID) platform on February 2018 were downloaded using the following selection criteria: i) origin: Asia, Europe and Africa; ii) host: avian; iii) collection date: 2005-2018 for H5N1 and 2013-2018 for H5NX; iv) minimum *HA* length of 1500 nt.

An initial phylogenetic tree was inferred using the neighbour-joining method available in MEGA 6 [1] to assign each sequence to a HPAI H5 genetic clade. The sequences were then separated into three distinct datasets according to the clade of belonging: *2.2*, *2.3.2.1c* and *2.3.4.4-B*.

For each virus, the following data were reported: virus name, subtype, host, collection date, sampling location. When specific epidemiological information were missing from GISAID, we performed a search in PubMed (https://www.ncbi.nlm.nih.gov/pmc/) to determine if the viruses were described in any paper. In particular, we looked for the following information: i) host species; ii) wild/domestic status; iii) place of collection; iv) exact collection date. Based on the available information we classified each virus sample in four host categories - Domestic Galliformes, Domestic Anseriformes, Wild Anseriformes, Wild Others - and nine geographic regions - Central Africa, West Africa, South Africa, East Europe, West Europe, Middle East, North-Central Asia, East Asia, South Asia.

Wild Anseriformes are classically recognized together with Charadiformes as the major natural reservoir of AIV [2]. However, given the lower prevalence of AIV infection in Charadiformes (about 1%) compared to ducks (about 10% with seasonal peaks of 20-60%) [2,3], and the higher number of available sequences from wild Anseriformes compared to the other orders, we decided to divide the host trait into four categories, each of which resulted to be well represented.

The geographical regions were defined as follows:

1. Central Africa: Democratic Republic of the Congo (DR Congo), Sudan, Uganda
2. West Africa: Burkina Faso, Cameroon, Ghana, Niger, Nigeria, Ivory Coast, Togo
3. South Africa
4. East Europe: Poland, Czech Republic, Croatia, Macedonia, Greece, Hungary, Bulgaria, Romania, Austria, Bosnia and Herzegovina, Slovenia, Tula, Krasnodar, Makhachkala, Domodedovo, Reshoty, Astrakhan, Adygea, Rostov, Tambov, Volgograd, Voronezh
5. West Europe: Sweden, Denmark, Germany, Belgium, Netherlands, Switzerland, France, UK, Italy
6. Middle East: Lebanon, Saudi Arabia, Egypt, United Arab Emirates
7. North-Central Asia: Mongolia, Inner Mongolia, Qinghai, Kazakhstan, Nuur Lake, Tyva, Chany, Kurgan, Siberia, Sartlan, Dovolnoe, Omsk, Novosibirsk

8. East Asia: Korea, Japan, Taiwan, Vietnam, Laos, Shandong, Jiangsu, Hunan, Zhejiang, Hubei, Whenzhou, Wuhnan, Yangzhou, Lianing, Shanghai, Heilongjiang, Shantou, Jiangxi, Liaoning
9. South Asia: India, Nepal, Bangladesh, Afghanistan, Bhutan, Tibet, Myanmar, Pakistan

Sequences for which it was not possible to retrieve sufficient epidemiological information (country of collection, host species, host status – domestic/wild, and collection year) were discarded from the dataset. We then added to each dataset African sequences obtained in our laboratory. Overall, clade *2.2* dataset contains 1514 sequences, *2.3.2.1c* dataset includes 621 sequences and clade *2.3.4.4-B* dataset contains 511 sequences.

Phylogenetic relationships were inferred for each of the datasets separately using the maximum likelihood (ML) method available in PhyML 3.1 [4], incorporating a general time-reversible (GTR) model of nucleotide substitution with a gamma-distributed (Γ) rate variation among sites and SPR branch swapping. Nodal supports were assessed using Shimodaira–Hasegawa (SH)-like branch supports.

In order to mitigate sampling bias, we applied three different subsampling procedures to the sequence data:

1) by selecting the samples based on epidemiological information (sampling location, collection date, host), in order to have roughly equitable numbers of sequences for each host and geographic category and year of collection. Among similarly categorized viruses, we selected the ones for which the most precise epidemiological information was available.

2) by selecting the sequences based on phylogenetic diversity, using the Phylogenetic Diversity Analyzer tool (www.cibiv.at/software/pda);

3) by randomly selecting the sequences.

The final datasets (Supplementary Data 1 to 9), each containing 240-260 sequences, were defined as follows:

- *2.2* Epi-based selection: 240 *HA* H5N1 subtype sequences from 2005-2011
- *2.2* Tree based selection: 240 *HA* H5N1 subtype sequences from 2005-2011
- *2.2* Random selection: 239 *HA* H5N1 subtype sequences from 2005-2011
- *2.3.2.1c* Epi-based selection: 259 *HA* H5N1 subtype sequences from 2009-2017
- *2.3.2.1c* Tree based selection: 240 *HA* H5N1 subtype sequences from 2009-2017
- *2.3.2.1c* Random selection: 234 *HA* H5N1 subtype sequences from 2009-2017
- *2.3.4.4-B* Epi-based selection: 247 *HA* multiple subtype sequences from 2013-2017
- *2.3.4.4-B* Tree based selection: 240 *HA* multiple subtype sequences from 2013-2017
- *2.3.4.4-B* Random selection: 240 *HA* multiple subtype sequences from 2013-2017

Clade *2.2*, and more specifically the sub-clades *2.2.1*, *2.2.1.1*, *2.2.1.1a* and *2.2.1.2* are endemic in Egypt since 2006 and are still circulating in the country. However, since the aim of this work is mainly to explore the means of virus spread into and within the African continent and these clades have been circulating only in Egypt where they are locally evolving (as clearly assessed by our ML phylogenetic analysis), we decide to include only data collected from 2005 to 2011. We considered this data sufficient to answer our research question.

African datasets

We collected all the *HA* sequences of African viruses available in GISAID or generated in our laboratory to create three African datasets, one for each clade.

All *HA* sequences and relative epidemiological information of the H5N1 and H5NX subtype available in the GISAID platform on February 2018 were downloaded using the following selection criteria: i) origin: Africa; ii) host: avian; iii) collection date: 2005-2008 for H5N1 subtype of clade *2.2*, 2014-2018 for H5N1 subtype of clade *2.3.2.1c*, and 2016-2018 for H5N8 for clade *2.3.4.4-B*; iv) minimum *HA* length of 1500 nt.

To each dataset we added the African sequences generated in our laboratory and few Eurasian representative sequences related to the African sequences, which based on the global phylogenetic analyses may be considered as the source of the African viruses.

These final datasets (Supplementary Data 10 to 12) resulted as follows:

- *2.2* Africa: 229 (196 from Africa) *HA* H5N1 subtype sequences from 2005-2008
- *2.3.2.1c* Africa: 221 (210 from Africa) *HA* H5N1 subtype sequences from 2014-2017
- *2.3.4.4-B* Africa: 103 (77 from Africa) *HA* H5N8 subtype sequences 2016-2018

For the same reason explained above, in the *2.2* Africa dataset we included only representative H5N1 Egyptian viruses collected from 2005 to 2008.

SUPPLEMENTARY FIGURES

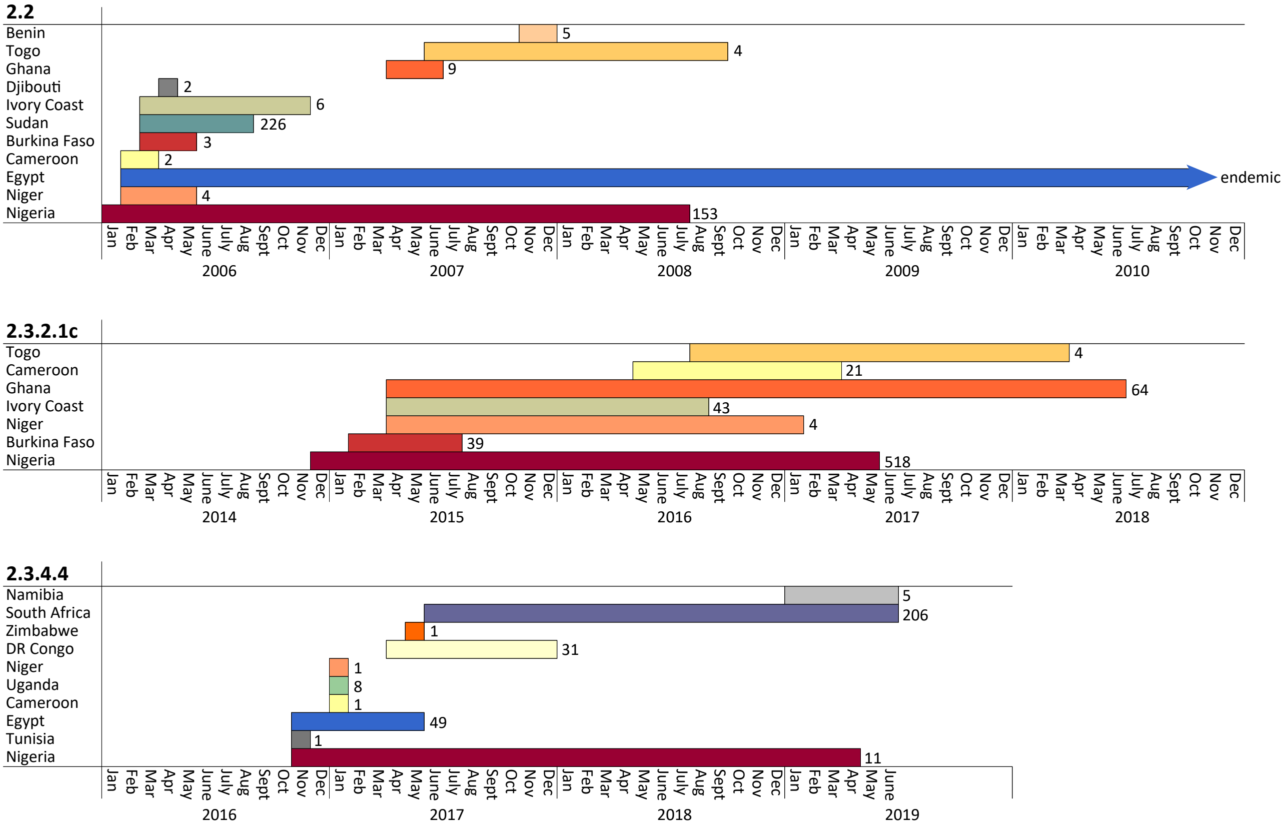

Supplementary Figure 1. **H5 HPAI outbreaks in Africa over time divided by clades.** The bars length represents the temporal extension of the epidemic (data from empres-i.fao.org, accessed July 25, 2019). The number of outbreaks for each country is shown on the right of the corresponding bar. The colours assigned to the different African countries reflect the colours used in Fig. 4.

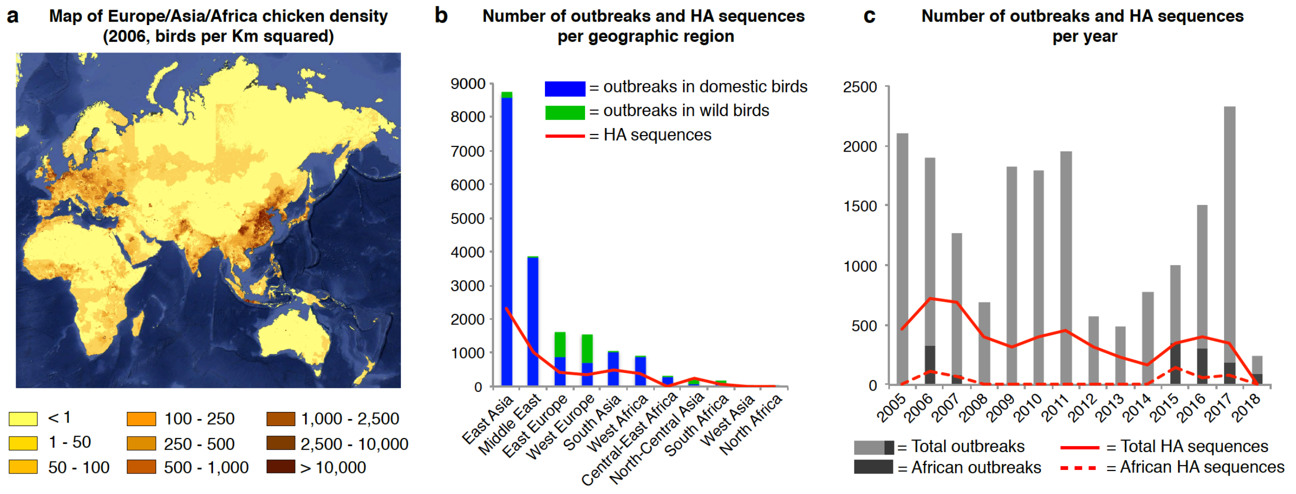

Supplementary Figure 2. **Poultry production, number of H5 outbreaks and H5 sequences.** (a) Map showing chicken density in Asia, Africa and Europe in 2006. The map was created from the Gridded Livestock of the World v2.0, (https://livestock.geo-wiki.org/ [5]). **(b)** Total number of outbreaks (bars) and *HA* sequences (red line) per geographic region available respectively from Empres-i (http://empres-i.fao.org) and GISAID (https://www.gisaid.org) for the H5N1 and H5N8 subtypes identified from 2005 to 2018 in wild (green) and domestic (blue) birds. **(c)** Total number of H5N1 and H5N8 outbreaks (grey bars) and *HA* sequences (red line) per year (2005 to 2018) available respectively from Empres-i and GISAID.

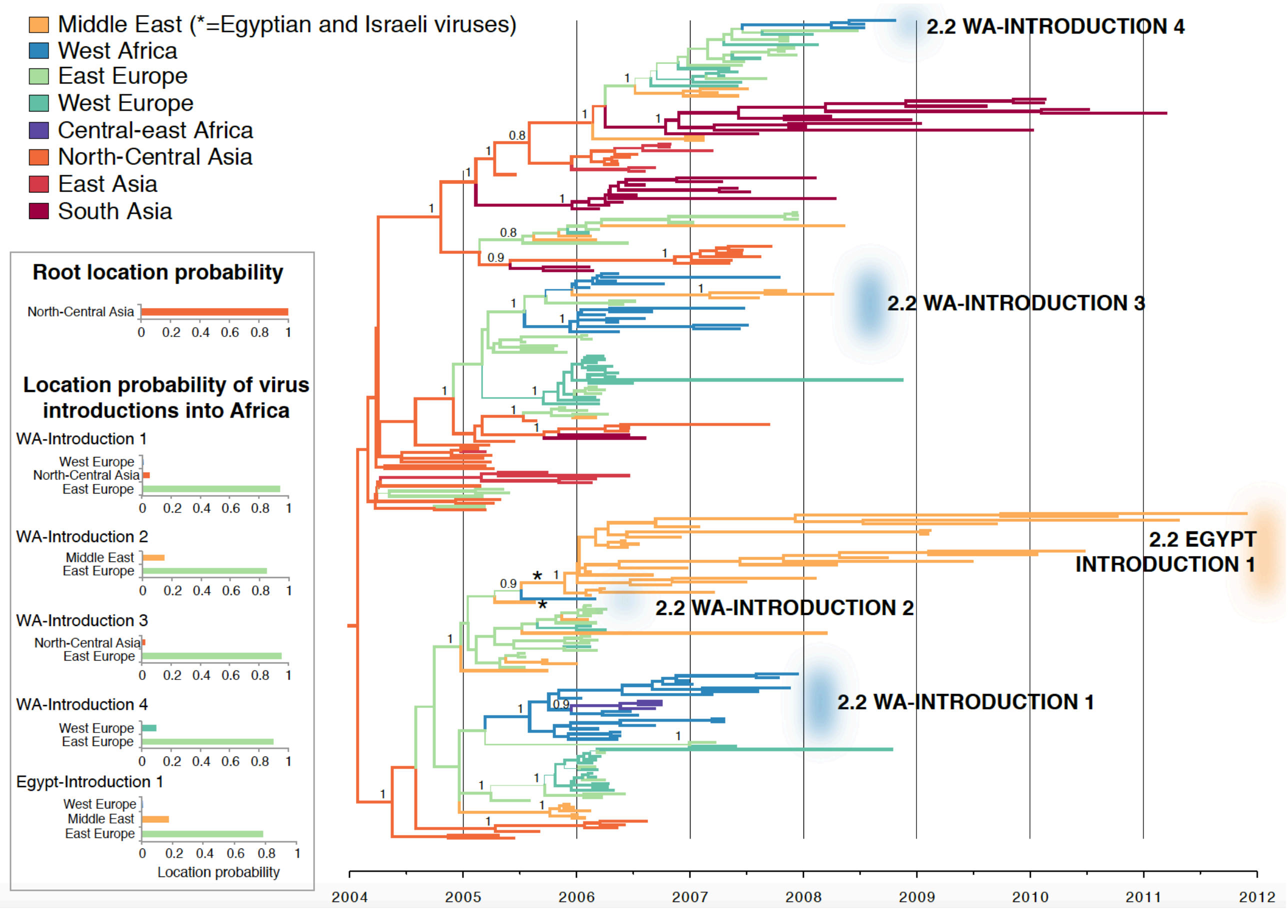

Supplementary Figure 3. Temporally structured MCC phylogenetic tree inferred for the *HA* segment of clade *2.2* – Global view. Branches are coloured according to the most probable area of origin. Branch thickness is proportional to the location posterior probability. Virus introductions into West Africa (WA) and the Middle East are indicated next to the branches. The location state posterior probability distributions for the root and the internal branches of each virus introduction into the African continent are shown in the graphs on the left.

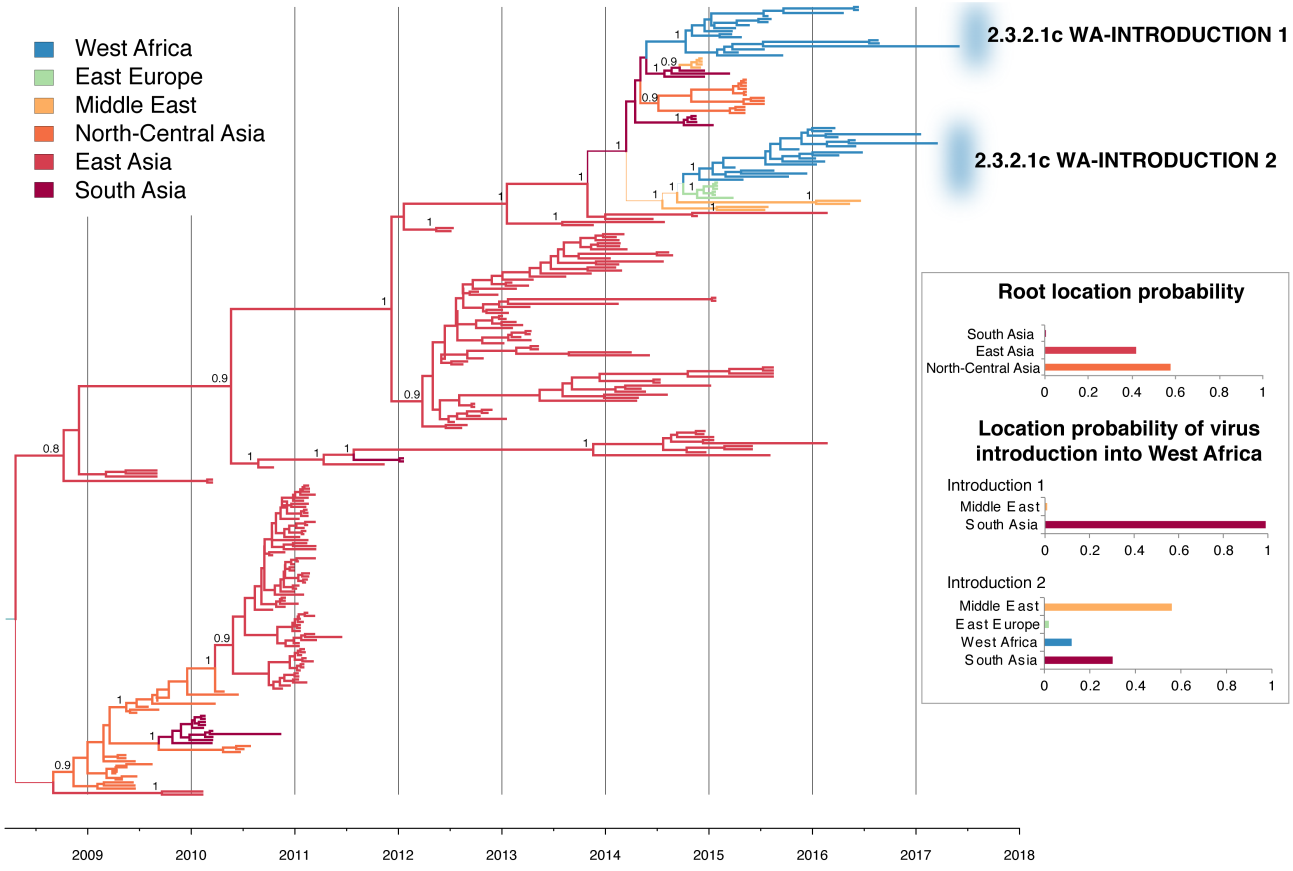

Supplementary Figure 4. Temporally structured MCC phylogenetic tree inferred for the *HA* segment of clade *2.3.2.1c* – Global view. Branches are coloured according to the most probable area of origin. Branch thickness is proportional to the location posterior probability. Virus introductions into West Africa (WA) are indicated next to the branches. The location state posterior probability distributions for the root and the internal branches of each virus introduction into the African continent are shown in the graphs on the right.

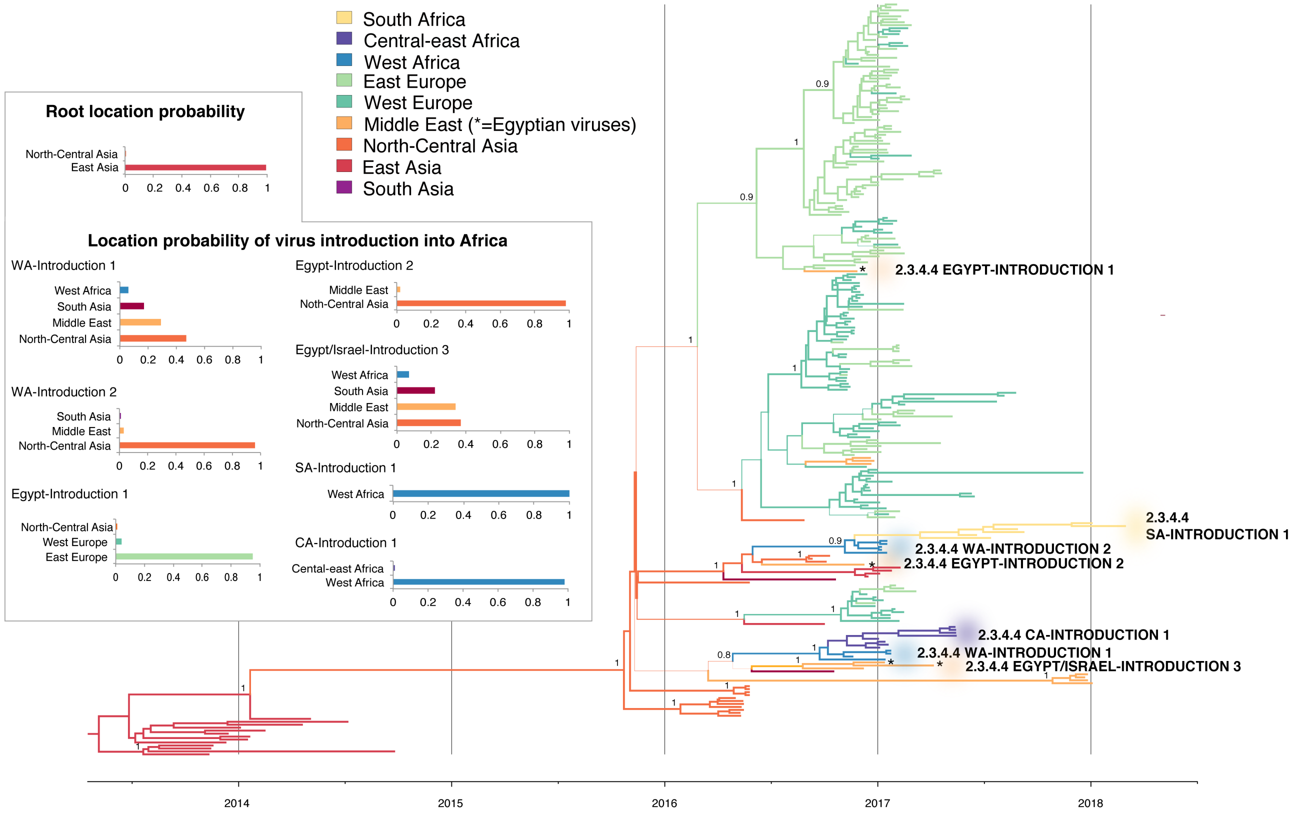

Supplementary Figure 5. Temporally structured MCC phylogenetic tree inferred for the *HA* segment of clade *2.3.4.4-B* – Global view. Branches are coloured according to the most probable area of origin. Branch thickness is proportional to the location posterior probability. Virus introductions into West Africa (WA), South Africa (SA), Central Africa (CA) and Egypt are indicated next to the branches. The location state posterior probability distributions for the root and the internal branches of each virus introduction into the African continent are shown in the graphs on the left.

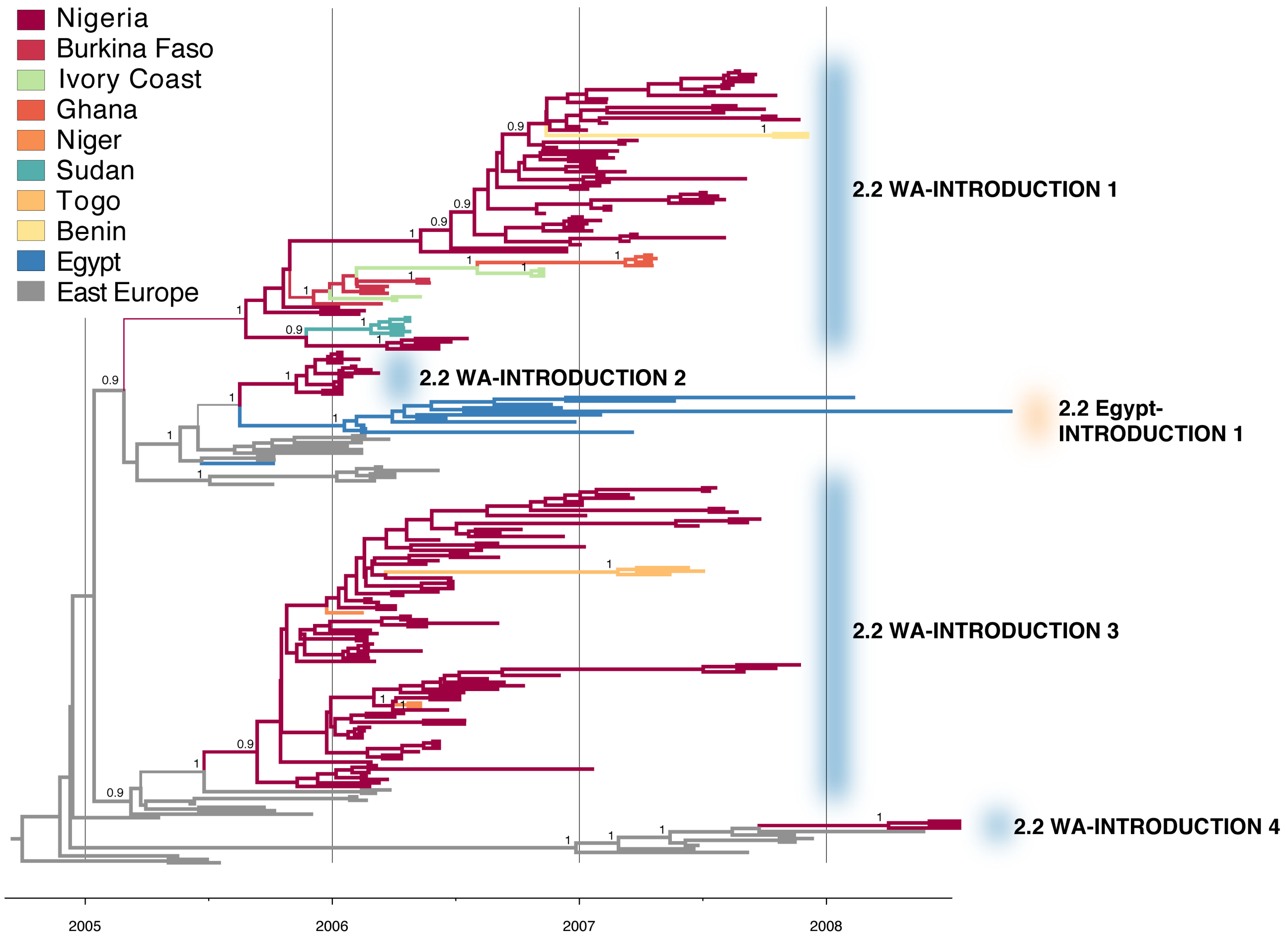

Supplementary Figure 6. Temporally structured MCC phylogenetic tree inferred for the *HA* segment of clade *2.2* – focus on the African continent. Branches are coloured according to the most probable African country of origin. Branch thickness is proportional to the location posterior probability. Viruses sampled outside the African continent are marked in black. Virus introductions into West Africa (WA) and Egypt are indicated next to the branches.

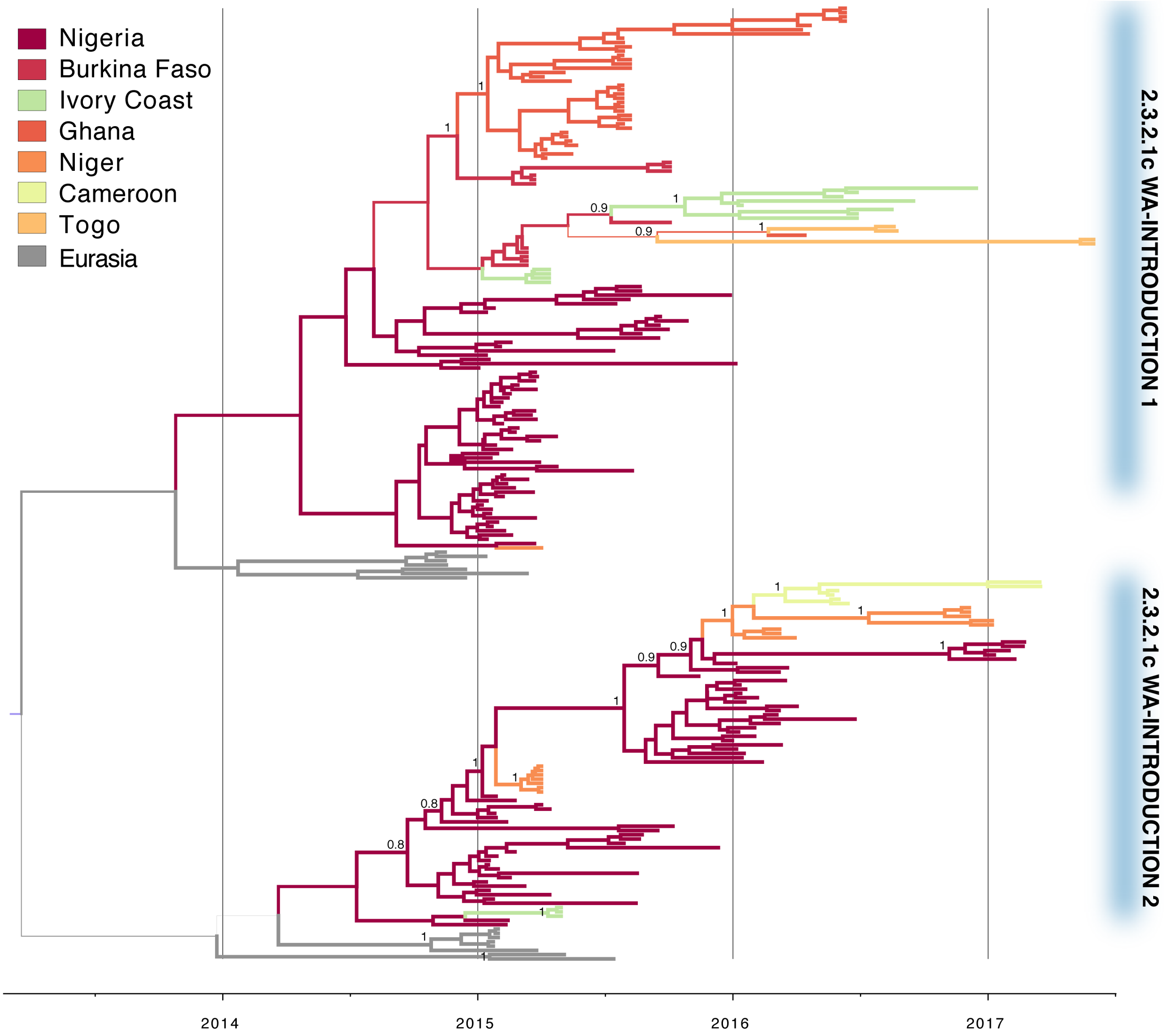

Supplementary Figure 7. **Temporally structured MCC phylogenetic tree inferred for the *HA* segment of clade *2.3.2.1c* – focus on the African continent.** Branches are coloured according to the most probable African country of origin. Branch thickness is proportional to the location posterior probability. Viruses sampled outside the African continent are marked in black. Virus introductions into West Africa (WA) are indicated next to the branches.

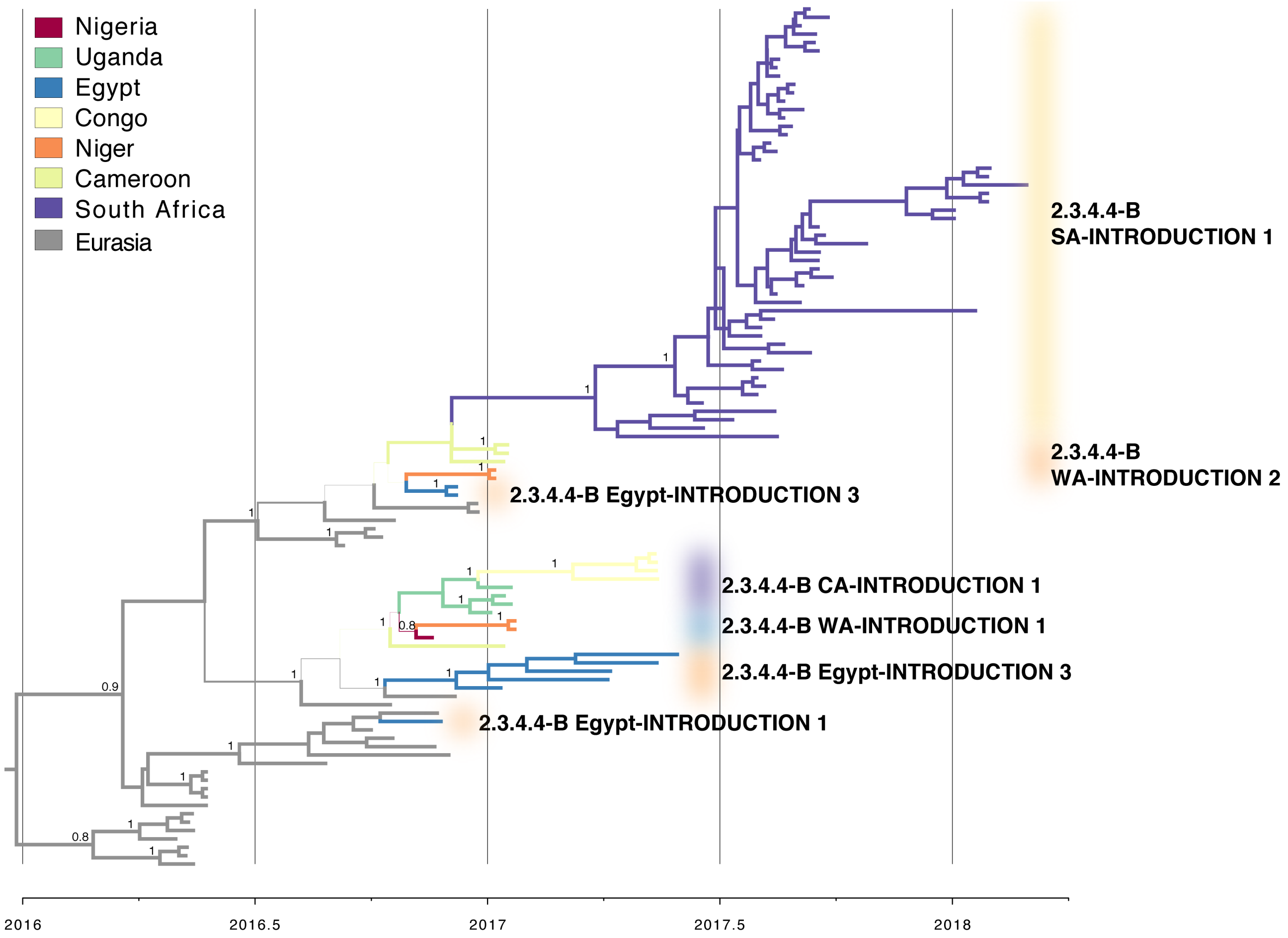

Supplementary Figure 8. **Temporally structured MCC phylogenetic tree inferred for the *HA* segment of clade *2.3.4.4-B* – focus on the African continent.** Branches are coloured according to the most probable African country of origin. Branch thickness is proportional to the location posterior probability. Viruses sampled outside the African continent are marked in black. Virus introductions into West Africa (WA), Egypt, South Africa (SA) and Central Africa (CA) are indicated next to the branches.

SUPPLEMENTARY TABLES

Supplementary Table 1. Bayes factor supports. Comparison of Bayes factor supports for individual transitions between discrete states inferred from the three distinct down-sampled datasets (Epi-based, tree-base and random) of each H5Nx clade. Bayes factors (BF) >5 and Posterior probability (PP) >0.5 are marked in bold. NC Asia – North-Central Asia; East Asia – E Asia; South Asia – S Asia; West Europe – West EU; East Europe – East EU; West Africa – WA; Central-East Africa – CE Africa; South Africa – SA; Middle East – ME.

**CLADE *2.2***		**EPI-BASED**		**TREE BASED**		**RANDOM**	
FROM	TO	BF	PP	BF	PP	BF	PP
NC Asia	E Asia	**>56512**	1.000	**18833.431**	1.000	**2562.772**	0.998
NC Asia	East EU	**>56512**	1.000	**50.353**	0.889	**100.968**	0.941
East EU	ME	**>56512**	1.000	**30.517**	0.829	**>56512**	1.000
East EU	West EU	**>56512**	1.000	**8.559**	0.577	**>56512**	1.000
NC Asia	S Asia	**1816.919**	0.997	**>56512**	1.000	**415.505**	0.985
West EU	East EU	**576.392**	0.989	**116.057**	0.949	2.903	0.316
WA	CE Africa	**493.890**	0.987	**493.890**	0.987	**93.402**	0.937
East EU	WA	**336.261**	0.982	**9.176**	0.594	**18833.431**	1.000
WA	ME	**6.895**	0.523	**8.037**	0.561	**18.199**	0.743
West EU	WA	0.369	0.055	**5.989**	0.488	1.490	0.192
NC Asia	West EU	0.204	0.031	**5.667**	0.474	0.494	0.073
**CLADE *2.3.2.1c***		**EPI-BASED**		**TREE BASED**		**RANDOM**	
FROM	TO	BF	PP	BF	PP	BF	PP
NC Asia	E Asia	**2740.710**	0.998	**19210.584**	1.000	**289.087**	0.985
S Asia	ME	**515.051**	0.992	**87.448**	0.953	**10.854**	0.718
E Asia	S Asia	**56.060**	0.929	**149.449**	0.972	0.983	0.187
NC Asia	S Asia	**25.637**	0.857	**62.565**	0.936	**187.879**	0.978
S Asia	NC Asia	**13.286**	0.757	**26.598**	0.862	2.586	0.377
S Asia	WA	**11.850**	0.735	**16.107**	0.790	3.914	0.478
ME	East EU	**6.507**	0.604	4.403	0.508	**16.998**	0.799
ME	WA	**6.344**	0.598	4.861	0.532	1.982	0.317
E Asia	NC Asia	2.501	0.369	0.529	0.110	**7.501**	0.637
**CLADE *2.3.4.4-B***		**EPI-BASED**		**TREE BASED**		**RANDOM**	
FROM	TO	BF	PP	BF	PP	BF	PP
East EU	West EU	**>65541**	1.000	**>65541**	1.000	**>65541**	1.000
West EU	East EU	**>65541**	1.000	**>65541**	1.000	**661.582**	0.989
WA	CE Africa	**71.313**	0.907	**105.151**	0.935	3.341	0.315
WA	SA	**45.923**	0.863	**20.551**	0.738	**46.534**	0.865
E Asia	NC Asia	**34.629**	0.826	**57.234**	0.887	**17.231**	0.703
NC Asia	ME	**24.231**	0.769	**53.978**	0.881	**13.081**	0.642
NC Asia	E Asia	**21.367**	0.746	2.716	0.272	**7.811**	0.517
NC Asia	West EU	**16.702**	0.696	**171.813**	0.959	**23.637**	0.764
NC Asia	S Asia	**15.383**	0.679	**5.933**	0.449	**7.880**	0.520
East EU	ME	**13.170**	0.644	2.894	0.284	2.473	0.254
NC Asia	WA	**6.416**	0.468	3.779	0.342	4.429	0.378
West EU	ME	3.947	0.352	**10.424**	0.589	**29.856**	0.804
ME	WA	4.329	0.373	**14.662**	0.668	2.209	0.233
ME	S Asia	4.117	0.361	**10.472**	0.590	2.679	0.269
CE Africa	WA	1.008	0.122	0.971	0.118	**20.132**	0.734
S Asia	CE Africa	1.312	0.153	1.382	0.160	**9.308**	0.561
WA	West EU	0.516	0.066	1.404	0.162	7.051	0.492

Supplementary Table 2. Time to most recent common ancestor (tMRCA). tMRCA for each virus introduction into the African continent estimated from the global and African datasets: West Africa (WA), Central Africa (CA), South Africa (SA), Egypt.

CLADE	VIRUS INTRODUCTION	Global dataset		African dataset	
		**Mean**	**95% HPD**	**Mean**	**95% HPD**
2.2	WA-Introduction 1	Mar 2005-Aug 2005	Apr 2004-Nov 2005	Mar 2005-Aug 2005	Mar 2005-Nov 2005
	WA-Introduction 2	July 2005-Apr 2006	Mar 2005-Apr 2006	Aug 2005-Nov 2005	May 2005-Dec 2005
	WA-Introduction 3	July 2005-Dec 2005	Mar 2005-Feb 2006	July 2005-Sept 2005	Nov 2004- Nov 2005
	WA-Introduction 4	June 2007-Mar 2008	Feb 2007-July 2008	Sept 2007-Mar 2008	June 2007-June 2008
	Egypt-Introduction 1	July 2005-Nov 2005	Mar 2005-Feb 2006	Aug 2005-Jan 2006	May 2005-Feb 2006
	CA-Introduction 1	Dec 2005-May 2006	Aug 2005-Sept 2006	Nov 2005-Feb 2006	Sept 2005-Mar 2006
2.3.2.1c	WA-Introduction 1	May 2014-Oct 2014	Feb 2014-Dec 2014	Oct 2013-Apr 2014	July 2013-Oct 2014
	WA-Introduction 2	Aug 2014-Nov 2014	June 2014-Jan 2015	Mar 2014-July 2014	June 2013-Sept 2014
2.3.4.4-B	WA-Introduction 1	Apr 2016-Sept 2016	Jan 2016-Nov 2016	Aug 2016-Sept 2016	July 2016-Nov 2016
	WA-Introduction 2	May 2016-Nov 2016	Jan 2016-Dec 2016	Sept 2016-Oct 2016	Sept 2016-Jan 2017
	Egypt-Introduction 1	Sept 2016-Nov 2016	July 2016-Nov 2016	Oct 2016-Nov 2016	Sept 2016-Nov 2016
	Egypt-Introduction 2	June 2016-Dec 2016	Mar 2016-Dec 2016	Oct 2016-Nov 2016	Sept 2016-Dec 2016
	Egypt-Introduction 3	Aug 2016-Nov 2016	May 2016-Dec 2016	Oct 2016-Nov 2016	Aug 2016-Jan 2017
	CA-Introduction 1	Oct 2016-Nov 2016	Aug 2016-Dec 2016	Oct 2016-Nov 2016	Sept 2016-Dec 2016
	SA-Introduction 1	Nov 2016-Mar 2017	Oct 2016-May 2017	Dec 2016-Mar 2017	Oct 2016-May 2017

SUPPLEMENTARY REFERENCES

1. Tamura, K., Stecher, G., Peterson, D., Filipski, A. & Kumar, S. MEGA6: Molecular Evolutionary Genetics Analysis Version 6.0. *Mol. Biol. Evol.* **30,** 2725–2729 (2013).

2. Olsen, B. *et al.* Global patterns of influenza a virus in wild birds. *Science* **312,** 384–8 (2006).

3. Munster, V. J. *et al.* Spatial, Temporal, and Species Variation in Prevalence of Influenza A Viruses in Wild Migratory Birds. *PLoS Pathog.* **3,** e61 (2007).

4. Guindon, S. *et al.* New Algorithms and Methods to Estimate Maximum-Likelihood Phylogenies: Assessing the Performance of PhyML 3.0. *Syst. Biol.* **59,** 307–321 (2010).

5. Robinson TP, *et al.* Mapping the Global Distribution of Livestock. PLoS ONE **9**(5): e96084. (2014). https://doi.org/10.1371/journal.pone.0096084
