# Supplementary material for: Disentangling the role of Africa in the global spread of H5 highly pathogenic avian influenza
Source: Nat Commun. 2019 Nov 22;10:5310. doi: 10.1038/s41467-019-13287-y (PMC6874648; doi:10.1038/s41467-019-13287-y)
Supplement: Supplementary file 3 — Reporting Summary [file 41467_2019_13287_MOESM3_ESM.pdf]

nature research

Corresponding author(s): Alice Fusaro
Isabella Monne

Last updated by author(s): Sep 19, 2019

Reporting Summary

Nature Research wishes to improve the reproducibility of the work that we publish. This form provides structure for consistency and transparency in reporting. For further information on Nature Research policies, see Authors & Referees and the Editorial Policy Checklist.

Statistics

For all statistical analyses, confirm that the following items are present in the figure legend, table legend, main text, or Methods section.

n/a	Confirmed	
☐	☒	The exact sample size (n) for each experimental group/condition, given as a discrete number and unit of measurement
☐	☒	A statement on whether measurements were taken from distinct samples or whether the same sample was measured repeatedly
☒	☐	The statistical test(s) used AND whether they are one- or two-sided<br>*Only common tests should be described solely by name; describe more complex techniques in the Methods section.*
☒	☐	A description of all covariates tested
☐	☒	A description of any assumptions or corrections, such as tests of normality and adjustment for multiple comparisons
☐	☒	A full description of the statistical parameters including central tendency (e.g. means) or other basic estimates (e.g. regression coefficient) AND variation (e.g. standard deviation) or associated estimates of uncertainty (e.g. confidence intervals)
☒	☐	For null hypothesis testing, the test statistic (e.g. F, t, r) with confidence intervals, effect sizes, degrees of freedom and P value noted<br>*Give P values as exact values whenever suitable.*
☐	☒	For Bayesian analysis, information on the choice of priors and Markov chain Monte Carlo settings
☒	☐	For hierarchical and complex designs, identification of the appropriate level for tests and full reporting of outcomes
☒	☐	Estimates of effect sizes (e.g. Cohen's d, Pearson's r), indicating how they were calculated

Our web collection on statistics for biologists contains articles on many of the points above.

Software and code

Policy information about availability of computer code

Data collection	ALIGNMENT OF INFLUENZA READS (OPEN SOURCE SOFTWARES):<br>FastQC v0.11.2: read quality assessment<br>scythe v0.991: trimming of Illumina adaptors<br>sickle v1.33: trimming of reads<br>BWA v0.7.12: alignment<br>Picard-tools v2.1.0 and GATK v3.5: correct potential errors, realign reads around indels with respect to the reference genome and recalibrate base quality<br>LoFreq v2.1.2: SNP calling
Data analysis	DATASET CONSTRUCTION AND GENETIC ANALYSES (OPEN SOURCE SOFTWARES):<br>Phylogenetic Diversity Analyzer v.1.0.3: sequence subsampling based on phylogenetic diversity<br>MAFFT v7: sequence alignment<br>BEAST v1.8.4: Bayesian analyses<br>Tracer v1.6: assessing the outputs of the Bayesian analyses<br>TreeAnnotator v1.8.4: generate maximum clade credibility trees<br>FigTree v1.4.2: visualize the trees<br>Spread D3 v0.9.6: Calculate Bayes factor support for individual transitions between discrete locations and visualize the spatial dynamics

For manuscripts utilizing custom algorithms or software that are central to the research but not yet described in published literature, software must be made available to editors/reviewers. We strongly encourage code deposition in a community repository (e.g. GitHub). See the Nature Research guidelines for submitting code & software for further information.

Data

Policy information about availability of data

All manuscripts must include a data availability statement. This statement should provide the following information, where applicable:

- Accession codes, unique identifiers, or web links for publicly available datasets
- A list of figures that have associated raw data
- A description of any restrictions on data availability

All datasets used for the analyses described in the study are available in the Supplementary Information files (Supplementary Data 1 to 12).
Accession numbers of the sequences downloaded from the public databases (GenBank or GISAID) or generated in this study are listed in the Supplementary Data 1 to 12.
Additional data are available from the corresponding authors upon request.

Field-specific reporting

Please select the one below that is the best fit for your research. If you are not sure, read the appropriate sections before making your selection.

☐ Life sciences ☐ Behavioural & social sciences ☒ Ecological, evolutionary & environmental sciences

For a reference copy of the document with all sections, see nature.com/documents/nr-reporting-summary-flat.pdf

Ecological, evolutionary & environmental sciences study design

All studies must disclose on these points even when the disclosure is negative.

Study description	The relevance of the African continent in the dynamics of the global spread of H5 highly pathogenic avian influenza (HPAI) viruses was investigated. To this aim we compared the spatiotemporal patterns of virus diffusion to/from and within Africa and investigated the role that poultry trade and wild bird migration may have played in spread of the H5 HPAI viruses collected during three epidemic waves (2005-2018).
Research sample	Hemagglutinin (HA) sequences of highly pathogenic avian influenza viruses of the H5 subtype of clade 2.2, 2.3.2.1c and 2.3.4.4-B collected between 2005 and 2018
Sampling strategy	All available sequences belonging to the clades under investigation
Data collection	All HA sequences and relavant epidemiological information of the H5N1 and H5NX subtype available in the public databases (GISAID or GenBank) were downloaded using the following selection criteria: i) origin: Asia, Europe and Africa; ii) host: avian; iii) collection date: 2005-2018 for H5N1 and 2013-2018 for H5NX; iv) minimum HA length of 1500 nt.<br>In addition, HA sequences of 38 H5 African viruses from Cameroon, Ghana, Ivory Coast, Niger, Nigeria and Togo were generated in this study.
Timing and spatial scale	Timing: from 2005 to 2018<br>Spatial scale: Africa, Asia and Europe. Details on the geographical regions analysed in the study are provided in the Supplementary material
Data exclusions	Low quality sequences containing frameshift indels were removed.<br>An initial phylogenetic tree was inferred using the neighbour-joining method to assign each sequence to a HPAI H5 genetic clade. Only sequences belonging to three clades responsible of trans-continental epidemic waves were used for the following analyses and separated into three distinct datasets: clade 2.2 (2005-2011, 1514 sequences); clade 2.3.2.1c (2009-2017, 621 sequences); and clade 2.3.4.4-B (2013-2018, 511 sequences).<br>Sequences for which were not possible to retrieve sufficient epidemiological information (country of collection, host species, host status – domestic/wild, and collection year) were discarded from the datasets.
Reproducibility	In this study we performed two main analyses: a global and a local (Africa-focused) analysis.<br>Global analyses – For each clade we performed discrete phylogeographic analyses on three distinct datasets obtained using three different subsampling strategies (epi-based, tree-based and random), for a total of nine datasets. A detailed description of each dataset is provided in the Supplementary Methods. The Bayes factors supporting all the migration events resulted from each analysis are reported in the Supplementary Table 1. The overall migration pattern is consistent across the down-sampling strategies, although some discrepancies between the datasets are indicative of important data gaps due to considerable differences in disease surveillance, outbreak reporting, sequencing efforts and data sharing among countries. This issue has been highlighted and discussed in the text.<br>The epi-based datasets, which have the most balanced distribution of samples among locations and hosts were also used to perform a more detailed phylogeographic reconstruction in continuous space. Results from discrete and continuous analyses showed a very similar pattern of virus diffusion.<br>Local analysis – For each clade we performed both a discrete and continuous phylogeographic analysis using all the available data. Results showed a very similar pattern of virus diffusion. In addition, results obtained from the Global datasets confirmed the overall virus spread within Africa obtained from the Local analyses.
Randomization	To assess robustness of our phylogeographic analyses and mitigate sampling bias, for the Global analysis we assembled three different datasets for each of the three H5Nx clade, each containing about 240-250 sequences using three different subsampling

	strategies for each dataset: 1) virus epidemiological information (sampling location, collection date, host) – epi-based dataset, 2) phylogenetic diversity – tree-based dataset, and 3) randomly down-sampling sequences – random dataset. A detailed description of the subsampling procedure is provided in the supplementary methods. The samples included in each of the nine datasets are provided in the Supplementary Data 1 to 9. Specifically, each table contains the list of the viruses, their HA accession numbers, and the relative epidemiological information.<br>Since subsamples of viral sequences used in the Local analyses are included in the Global analyses, subsampling and randomization are not specifically done for the Local datasets.
Blinding	No blinding was performed

Did the study involve field work? ☐ Yes ☒ No

Reporting for specific materials, systems and methods

We require information from authors about some types of materials, experimental systems and methods used in many studies. Here, indicate whether each material, system or method listed is relevant to your study. If you are not sure if a list item applies to your research, read the appropriate section before selecting a response.

Materials & experimental systems

n/a	Involved in the study	
☒	☐	Antibodies
☒	☐	Eukaryotic cell lines
☒	☐	Palaeontology
☒	☐	Animals and other organisms
☒	☐	Human research participants
☒	☐	Clinical data

Methods

n/a	Involved in the study	
☒	☐	ChIP-seq
☒	☐	Flow cytometry
☒	☐	MRI-based neuroimaging
