# Supplementary material for: Disentangling the role of Africa in the global spread of H5 highly pathogenic avian influenza
Source: Nat Commun. 2019 Nov 22;10:5310. doi: 10.1038/s41467-019-13287-y (PMC6874648; doi:10.1038/s41467-019-13287-y)
Supplement: Supplementary file 4 — Description of Additional Supplementary Files [file 41467_2019_13287_MOESM4_ESM.pdf]

Description of Additional Supplementary Files

File Name: Supplementary Data 1
Description: List of H5N1 virus sequences belonging to clade 2.2, collected globally and selected based on epidemiological information (2.2 Epi-based dataset). For each sequence, GenBank or GISAID accession number of the HA gene, virus name, subtype, clade, location of sampling and the assigned discrete location trait, host order and assigned discrete host trait, collection date, and submitting and originating laboratory (for GISAID sequences only) are provided.

File Name: Supplementary Data 2
Description: List of H5N1 virus sequences belonging to clade 2.2, collected globally and selected based on phylogenetic diversity (2.2 Tree based dataset). For each sequence, GenBank or GISAID accession number of the HA gene, virus name, subtype, clade, location of sampling and the assigned discrete location trait, host order and assigned discrete host trait, collection date, and submitting and originating laboratory (for GISAID sequences only) are provided.

File Name: Supplementary Data 3
Description: List of H5N1 virus sequences belonging to clade 2.2, collected globally and randomly selected (2.2 random dataset). For each sequence, GenBank or GISAID accession number of the HA gene, virus name, subtype, clade, location of sampling and the assigned discrete location trait, host order and assigned discrete host trait, collection date, and submitting and originating laboratory (for GISAID sequences only) are provided.

File Name: Supplementary Data 4
Description: List of H5N1 virus sequences belonging to clade 2.3.2.1c, collected globally and selected based on epidemiological information (2.3.2.1c Epi-based dataset). For each sequence, GenBank or GISAID accession number of the HA gene, virus name, subtype, clade, location of sampling and the assigned discrete location trait, host order and assigned discrete host trait, collection date, and submitting and originating laboratory (for GISAID sequences only) are provided. Viral sequences originated in this study are indicated.

File Name: Supplementary Data 5
Description: List of H5N1 virus sequences belonging to clade 2.3.2.1c, collected globally and selected based on phylogenetic diversity (2.3.2.1c Tree based dataset). For each sequence, GenBank or GISAID accession number of the HA gene, virus name, subtype, clade, location of sampling and the assigned discrete location trait, host order and assigned discrete host trait, collection date, and submitting and originating laboratory (for GISAID sequences only) are provided. Viral sequences originated in this study are indicated.

File name: Supplementary Data 6
Description: List of H5N1 virus sequences belonging to clade 2.3.2.1c, collected globally and randomly selected (2.3.2.1c random dataset). For each sequence, GenBank or GISAID accession number of the HA gene, virus name, subtype, clade, location of sampling and the assigned discrete location trait, host order and assigned discrete host trait, collection date, and submitting and originating laboratory (for GISAID sequences only) are provided. Viral sequences originated in this study are indicated.

File Name: Supplementary Data 7
Description: List of H5Nx virus sequences belonging to clade 2.3.4.4-B, collected globally and selected based on epidemiological information (2.3.4.4-B Epi-based dataset). For each sequence, GenBank or GISAID accession number of the HA gene, virus name, subtype, clade, location of

sampling and the assigned discrete location trait, host order and assigned discrete host trait, collection date, and submitting and originating laboratory (for GISAID sequences only) are provided. Viral sequences originated in this study are indicated.

File Name: Supplementary Data 8
Description: List of H5Nx virus sequences belonging to clade 2.3.4.4-B, collected globally and selected based on phylogenetic diversity (2.3.4.4-B Tree based dataset). For each sequence, GenBank or GISAID accession number of the HA gene, virus name, subtype, clade, location of sampling and the assigned discrete location trait, host order and assigned discrete host trait, collection date, and submitting and originating laboratory (for GISAID sequences only) are provided. Viral sequences originated in this study are indicated.

File Name: Supplementary Data 9
Description: List of H5Nx virus sequences belonging to clade 2.3.4.4-B, collected globally and randomly selected (2.3.4.4-B random dataset). For each sequence, GenBank or GISAID accession number of the HA gene, virus name, subtype, clade, location of sampling and the assigned discrete location trait, host order and assigned discrete host trait, collection date, and submitting and originating laboratory (for GISAID sequences only) are provided. Viral sequences originated in this study are indicated.

File Name: Supplementary Data 10
Description: List of all available African H5N1 virus sequences and related Eurasian sequences belonging to clade 2.2 (2.2 Africa dataset). For each sequence, GenBank or GISAID accession number of the HA gene, virus name, subtype, clade, location of sampling and the assigned discrete location trait, collection date, and submitting and originating laboratory (for GISAID sequences only) are provided.

File Name: Supplementary Data 11
Description: List of all available African H5N1 virus sequences and related Eurasian sequences belonging to clade 2.3.2.1c (2.3.2.1c Africa dataset). For each sequence, GenBank or GISAID accession number of the HA gene, virus name, subtype, clade, location of sampling and the assigned discrete location trait, collection date, and submitting and originating laboratory (for GISAID sequences only) are provided. Viral sequences originated in this study are indicated.

File Name: Supplementary Data 12
Description: List of all available African H5N8 virus sequences and related Eurasian sequences belonging to clade 2.3.4.4-B (2.3.4.4-B Africa dataset). For each sequence, GenBank or GISAID accession number of the HA gene, virus name, subtype, clade, location of sampling and the assigned discrete location trait, collection date, and submitting and originating laboratory (for GISAID sequences only) are provided. Viral sequences originated in this study are indicated.
